# TAB2 Promotes the Stemness and Biological Functions of Cervical Squamous Cell Carcinoma Cells

**DOI:** 10.1155/2021/6550388

**Published:** 2021-06-30

**Authors:** Yijia Zhou, Yuandong Liao, Chunyu Zhang, Junxiu Liu, Wei Wang, Jiaming Huang, Qiqiao Du, Tianyu Liu, Qiaojian Zou, Hua Huang, Pan Liu, Shiyin Ooi, Run Chen, Meng Xia, Hongye Jiang, Manman Xu, Yuwen Pan, Shuzhong Yao

**Affiliations:** ^1^Department of Obstetrics and Gynecology, The First Affiliated Hospital, Sun Yat-sen University, Guangzhou, 510080 Guangdong, China; ^2^Department of Obstetrics and Gynecology, Northwest Women's and Children's Hospital, Xi'an, Shaanxi, China

## Abstract

Cancer stem cells are a key population participating in the promotion of the cervical cancer progression through interacting with cancer cells. Existing studies have preliminary revealed that cervical cancer stem cells contribute to tumor recurrence and chemotherapy resistance. However, the specific mechanisms involved in regulating cell functions remain largely unknown. Here, we analyzed published data from public databases and our global transcriptome data, thus identifying cancer-related signaling pathways and molecules. According to our findings, upregulated TAB2 was correlated to stem cell-like properties of cervical cancer. Immunohistochemistry staining of TAB2 in normal and cervical cancer tissues was performed. The cell function experiments demonstrated that knockdown of TAB2 reduced the stemness of cervical cancer cells and, importantly, prevented cervical cancer progression. Collectively, the therapeutic scheme targeting TAB2 may provide an option for overcoming tumor relapse and chemoresistance of cervical cancer via obstructing stemness maintenance.

## 1. Introduction

Cervical cancer (CC) is one of the leading causes for cancer mortality in females in developing countries, with an annual incidence of 560,000 new cases and 300,000 deaths worldwide in 2018 [1]. Although patients with early-stage CC usually have a good prognosis, it can be deteriorated by recurrent or metastatic disease. For advanced CC, cisplatin treatment-based chemotherapy is preferred, although its efficacy is unsatisfactory with a low response rate [2]. Recently, combined therapies for advanced CC are demonstrated to achieve a better overall survival (OS) and response rate [3, 4].

Recent advances have highlighted the existence of a group of cells in tumors that are highly plastic and self-renewing, which are known as cancer stem cells (CSCs). CSCs are featured by the ability to differentiate into tumor cell populations in response to stimuli from the surrounding microenvironment [5]. They share the same characteristics as normal stem cells, such as self-renewal, migration, differentiation, and avoidance of apoptosis. Emerging studies have confirmed the vital role of CSCs in oncogenesis and progression, which are capable of determining tumor progression rate, metastasis, tumor chemotherapy resistance, and disease prognosis. Therefore, CSCs are associated with poor prognosis. So far, CSCs have been detected in many types of cancer [6, 7], including but not limited to cervical [8]. Notably, several studies have found that several stem cell-related genes are closely associated with tumorigenesis. Previous findings have shown the critical functions of SOX2 and BMI1 in the carcinogenesis of CC [9]. Prior studies have suggested that targeted treatment of molecules in CSCs is important in cancer [10]. Thus, it is of great significance to search for molecules regulating the stemness of cervical cancer stem cells in CC.

In this study, we examined the expression pattern in Siha-derived cancer stem-like cells (Siha sphere) and Siha cell line by RNA sequencing. It is found that transforming growth factor *β*-activated kinase 1 binding protein 2 (TAB2) was significantly upregulated in Siha-derived cancer stem-like cells. TAB2 level was associated with the OS of CC in TCGA database. Knockdown of TAB2 in Siha and MS751 cell lines significantly inhibited the proliferation and induced apoptosis in vitro. Further studies confirmed that TAB2 promoted tumorigenesis and development of CC via modulating CSCs. Taken together, the present study revealed an undefined role of TAB2 in CC, providing both a prognostic marker and drug candidate for this malignancy.

## 2. Method

### 2.1. Cell Culture and Patient Samples

The human CC cell lines Siha and HeLa and the normal human cervical H8 cell line were purchased from the Type Culture Collection of the Chinese Academy of Sciences. Cells were cultured in Dulbecco's Modified Eagle's Medium (DMEM, Gibco, 11885-076) with 10% fetal bovine serum (FBS, Gibco, 10270-106) and 1% penicillin-streptomycin (Gibco, 15140122) at 37°C with 5% CO_2_. MS751 cell line was cultured in MEM (Gibco, 11095080) supplemented with 10% FBS and 1% nonessential amino acid (Gibco, 11140-050).

All the tissues were collected from CC patients who underwent surgery in the First Affiliated Hospital, Sun Yat-sen University. Tumor samples were fixed in 4% formalin overnight at room temperature and embedded in paraffin. Informed written consent was obtained from all patients prior to the study.

### 2.2. Cell Transfection

TAB2 siRNAs were constructed and their sequences were listed as follows: hs-TAB2-si-1: GGUGCAUGUUACAGAAUAAdTdT (sense) and UUAUUCUGUAACAUGCACCdTdT (antisense); hs-TAB2-si-2: CAGCAUUAGUGAUGGACAAdTdT (sense) and UUGUCCAUCACUAAUGCUGdTdT (antisense).

### 2.3. RT-qPCR

Cellular total RNA was extracted according to the instructions of the TRIzol reagent (Invitrogen, 15596026). Then, 1 *μ*g RNA was utilized to reverse transcribe to cDNA by the standard procedure of the PrimeScript RT master mix kit (TaKaRa, RR036A). After that, TB Green™ Premix Ex Taq II kit (TaKaRa, RR820B) was utilized for real-time PCR and operated on the Bio-Rad CXF96 real-time system (Bio-Rad, USA). GAPDH was selected as the internal reference to calculate the relative level. The primer pairs were listed as follows: TAB2 (forward): 5′-GGACCTGCCCTGGAAAAGAA-3′, TAB2 (reverse): 5′-TAGCTGTTCTTGGTTGGCCC-3′; GAPDH (forward): 5′-TGTGGGCATCAATGGATTTGG-3′, GAPDH (reverse): 5′-ACACCATGTATTCCGGGTCAAT-3′.

### 2.4. Western Blotting

Cellular protein of CC was obtained with ice-cold RIPA lysis solution, and Pierce™ BCA Protein Assay Kit (Thermo Fisher Scientific, 23227) was used to detect protein concentration in cell lysates. Then, the protein was dissolved in loading buffer and high-temperature denatured for 10 minutes. After separating by 10% SDS polyacrylamide gel electrophoresis, the protein was transferred to the PVDF membranes (Millipore, Germany). The membrane was blocked for 1 h prior to overnight incubation of primary antibodies (anti-TAB2, Proteintech, 14410-1-AP-50 *μ*l; anti-BMI1, Abcam, ab14389-25 *μ*g; anti-SOX2, Abcam, ab97959-100 *μ*g; and anti-GAPDH, Abcam, ab70699) at 4°C, followed by secondary antibody incubation at room temperature for 1 h on the other day. The protein-antibody complex was detected with enhanced chemiluminescence (ECL) reagent.

### 2.5. Colony Formation Assay

Siha or MS751 cells were seeded in the 6-well plate (2 × 10^3^ cells/well). After 14 days, the cells were fixed with 4% paraformaldehyde for 30 minutes and stained with 0.1% crystal violet, followed by capturing cell colonies. Each group was repeated in triplicate.

### 2.6. Cell Proliferation Assay

CCK-8 assay (Dojindo, CCK8-500) was performed to measure cell proliferation. According to the standard procedure, the cell suspension containing 2 × 10^3^ cells (100 *μ*l) was seeded into each well of the 96-well plate and cultured at 37°C. At indicated time points, 10 *μ*l of reaction agent was added to each well and incubated at 37°C for 1 hour, followed by detecting the absorbance at 450 nm wavelength (Infinite 200 PRO).

### 2.7. Cell Migration Assay

Cell migration ability was detected via Transwell assay. Cells were seeded in the Transwell insert (Merck Millibo, MCHT06H48) at 1 × 10^4^/ml. DMEM containing 20% FBS was applied in a 24-well plate where Transwell insert was placed. After incubation at 37°C with 5% CO_2_ for 24 h, the samples were fixed with 4% paraformaldehyde at 4°C for 30 minutes. After washing with PBS, migratory cells were stained with crystal violet and incubated at room temperature for 45 minutes. Then, cells on the upper side of the insert were removed with a cotton swab and rinsed thoroughly with PBS. The average number of cells in 3 randomly selected view per sample was calculated.

### 2.8. Flow Cytometric Analysis of Annexin V Apoptosis and Cell Cycle Progression

Apoptosis rate was determined by the Annexin V Apoptosis Detection Kit (Dojindo, AD10). Briefly, CC cells were seeded into 6-well plates (1 × 10^5^/ml/well) for apoptosis analysis and cultured in complete medium at 37°C for two days. Then, cells were collected and stained with the kit after treatment with non-EDTA trypsin. CC cells used for determining cell cycle progressions were transplanted into a 6-well plate (5 × 10^5^ ml/well) and cultured at 37°C overnight. After digesting in non-EDTA trypsin, cells were fixed overnight with 75% ethanol and stained with the kit (MultiScience, 70-CCS012) for 15 minutes on the next day. Both experiments were operated on a flow cytometer with BD FACSDiva software 6.0 (BD Biosciences, New York, NY, USA) in triplicate.

### 2.9. Flow Cytometric Analysis of Aldehyde Dehydrogenase Activity

Acetaldehyde dehydrogenase activity was determined using the ALDEFLUOR fluorescent kit (STEM Cell Technology, 01700). In brief, a test tube and a control tube were labeled for each sample. After collecting cells in a test tube, an activated fluorinated reagent was added in cell suspension. Then, half of the suspension and 5 *μ*l of diethylaminobenzaldehyde (DEAB) were added in the control tube and incubated for half an hour at 37°C. The supernatant was removed, and precipitant was resuspended in the determination buffer for analysis of aldehyde dehydrogenase activity using flow cytometry.

### 2.10. Immunohistochemistry (IHC) Staining

CC sections were dewaxed in xylene and then rehydrated in ethanol with gradient concentrations. After PBS rinsing, the sections were incubated in 0.01 mol/l sodium citrate buffer (pH 6.0) and antigen retrieval was performed at a high temperature. After blocking the endogenous peroxidases in 3% H_2_O_2_ at room temperature for 10 minutes, CC sections were incubated with anti-TAB2 (1 : 200, Proteintech, 14410-1-AP-50 *μ*l) and anti-SOX2 (1 : 200, Abcam, ab97959-100 *μ*g) at 4°C overnight. Then, they were incubated with horseradish peroxidase- (HRP-) labeled rabbit-goat at room temperature for 1 h. Positive expressions of TAB2 and SOX2 were determined by 3,3′-diaminobenzidine (DAB) staining solution.

The intensity of IHC staining (*I*) was scored as 0 (negative), 1 (weak), 2 (medium), or 3 (strong) grades. The percentage of positively stained cells (*P*) was scored as 0 (negative), 1 (≤10%), 2 (11%-50%), 3 (51%-75%), or 4 (>75%) grades. IHC score (*Q*) = *P* × *I*, in which *P* was the percentage of positively cells and *I* was the intensity of IHC staining.

### 2.11. RNA Sequencing

Siha cells and Siha sphere were sent for RNA sequencing. In short, total RNA of each sample was collected in 1 ml of TRIzol for further analysis. Then, the quality and purity of total RNA were detected by Nano-300. According to Illumina protocol, cDNA libraries were constructed from RNA samples for performing Illumina paired-end (PE) sequencing on the Illumina HiSeq 2000 (Illumina, San Diego, CA) platform. Paired-end reads (2100 bp) were produced at Noggin Bioinformatics Co., Ltd. (Beijing, China).

### 2.12. Sphere Formation Assays

Following the previous method [11], the stem cell medium containing 3 × 10^3^ cells/ml was placed into the 6-well ultralow plate and incubated for 1-2 weeks at 37°C with 5% CO_2_. The stem cell media medium should be replaced every two days. After the formation of spheres, they were digested into individual spheres with 0.25% trypsin-EDTA and captured under a microscope for counting. After six generations of sphere formation, we obtained a group of cells with greater stemness, named Siha sphere. Spherical cell clusters larger than 75 *μ*m in diameter are considered to be spheres formed by self-renewal of CSCs.

### 2.13. Statistical Analysis

The unpaired Student *t*-test was used to determine statistical significance. Data was expressed as mean ± standard deviation. Mathematical analysis of all the results was carried out by GraphPad Prism 8.0.

## 3. Results

### 3.1. Siha Sphere Displays Elevated CSC-Like Properties

**Figure 1:**
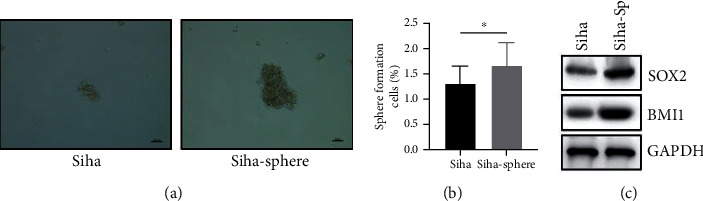
The ability of cell stemness maintenance in Siha sphere is significantly higher than that in parental cells. (a) The sphere formation ability was significantly enhanced in the Siha sphere group compared with the Siha group. (b) The enrichment of tumor stem cells significantly enhanced sphere formation efficiency in the Siha sphere group compared with the Siha group. (c) Western blot was performed to detect protein levels of SOX2 and BMI1 in Siha cells and Siha sphere.

Previous studies have shown that CSCs can be selectively enriched in tumor cells [12, 13]. Tumor sphere formation experiment has been considered as an ideal method to detect the characteristic of cell self-renewal. CC cells seeded in serum-free medium were optimal for the growth of tumor spheres. We compared the sphere-forming efficiency of the sixth-generation sphere with the original cell line and proved that CSCs with a higher sphere-forming efficiency were enriched in CC cells (Figures 1(a) and 1(b)). Consistent results were found in the subsequent Western blotting assay as higher protein levels of BMI1 and SOX2 were detected in Siha sphere than in Siha cells (Figure 1(c)).

### 3.2. TAB2 Is Related to Cancer Cell Stemness

**Figure 2:**
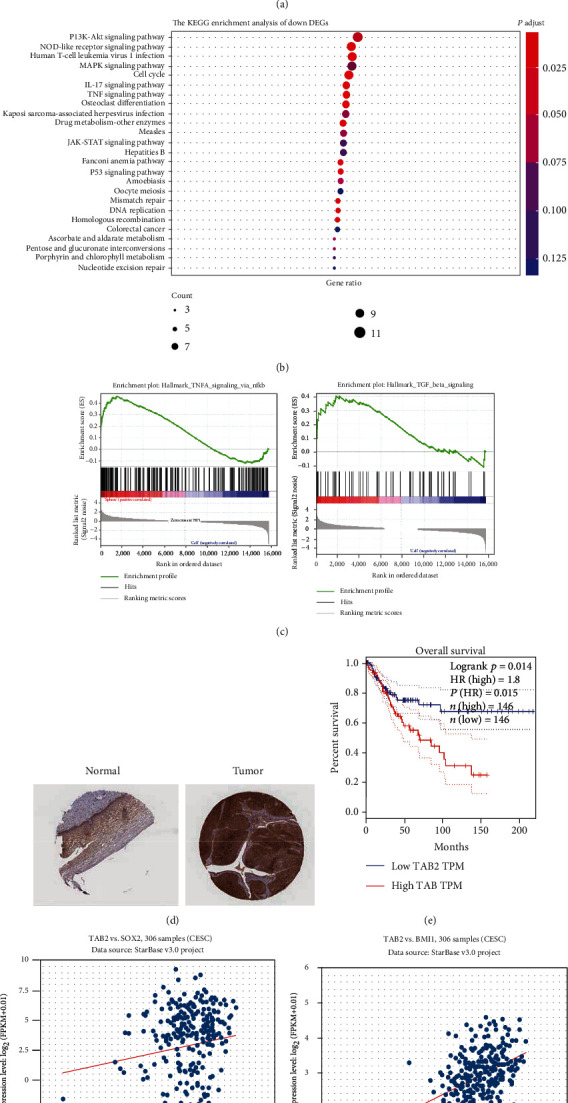
The next-generation sequencing and bioinformation analysis. (a) The DEGs were displayed by volcano scatter plots. (b) The KEGG enrichment analysis of DEGs. (c) GSEA showing the related pathway regulated by TAB2. (d) The Human Protein Atlas dataset presenting the expression of TAB2 in normal and cervical cancer tissues. (e) Kaplan-Meier analysis of overall survival in patients with cervical cancer (*n* = 292). Patients were divided into low and high group according to the cut-off level of TAB2 in TCGA database. (f) Coexpression analysis of TAB2 and SOX2 based on the data from StarBase. (g) Coexpression analysis between TAB2 and BMI1 according to the data from StarBase.

To investigate the molecular mechanism of CSCs in regulating the progression of CC, RNA sequencing was performed to determine the downstream mechanisms in Siha sphere and Siha cells. Differentially expressed genes (DEGs) were divided into upregulation, not significant (non-sig.), and downregulation sets depicted by the volcano scatter plots (Figure 2(a)). KEGG enrichment analysis showed that downregulated genes were selectively enriched in a series of stemness regulatory pathways, tumorigenesis, and development-related pathways; among which, the PI3K-AKT signaling pathway was found to be the most enriched (Figure 2(b)). In addition, some representative oncogenic features, such as the tumor necrosis factor (TNF) signal transduction and transforming growth factor-*β*, were statistically enriched in Siha sphere, suggesting that CSCs may regulate tumorigenesis, proliferation, metastasis, and chemotherapy resistance of CC (Figure 2(c)). TAB2 was screened out as a downstream molecule in the highly expressed gene set, because it was involved in both pathways. The Human Protein Atlas data showed that TAB2 protein was upregulated in CC tissues compared with normal tissues (Figure 2(d)). Subsequently, it was found that TAB2 was significantly negatively correlated with the overall survival of CC patients in The Cancer Genome Atlas (TCGA) database (Figure 2(e)). The StarBase database was used to analyze the coexpression of TAB2 and classical stemness molecules (BMI1 and SOX2) in 306 cervical squamous cell carcinoma samples. It was found that the expression level of TAB2 was significantly positively correlated with that of BMI1 in cervical squamous cell carcinoma samples, which was also consistent with previous data (Figure 2(f)).

### 3.3. TAB2 and SOX2 Are Overexpressed in CC Patients

**Figure 3:**
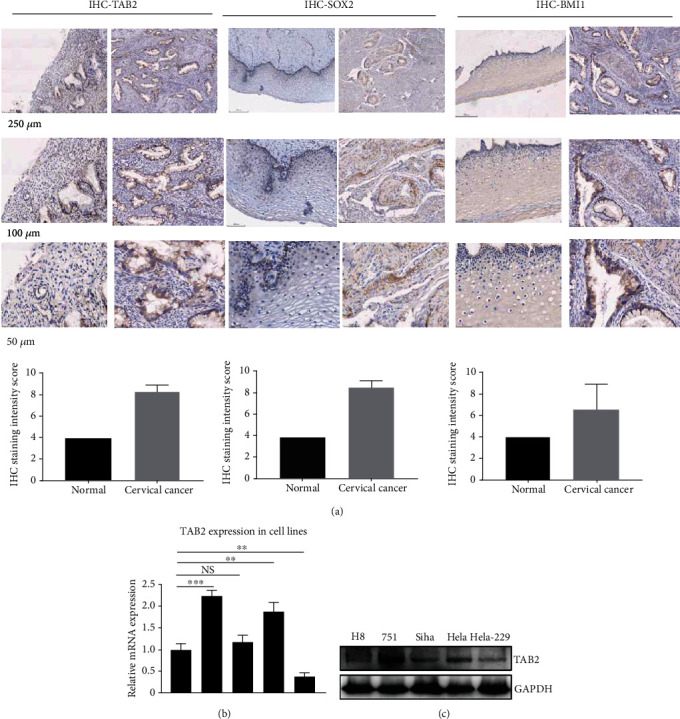
The expression of TAB2 in cervical cancer. (a) IHC staining showing that positive expressions of TAB2 and SOX2 were significantly high in cervical cancer. (b) The mRNA expression of TAB2 in cervical cancer cell lines was tested by qPCR experiment. (c) The protein level of TAB2 in different cervical cancer cell lines.

To further verify the regulatory mechanism of TAB2 in regulating the characteristics of CSCs in CC, positive expressions of TAB2 and stemness marker molecules in CC patients in vivo were examined by IHC staining. Since BMI1 and SOX2 are major markers of CCSCs, we investigated their expression patterns in CC sections. Positive expressions of TAB2, BMI1, and SOX2 were all significantly higher in CC sections compared with those of normal tissues (Figure 3(a)). For further clarifying the function of TAB2 in vitro, we selected 4 CC cell lines and 1 normal cell line to determine the expression level of TAB2 by Western blotting and RT-qPCR assay (Figures 3(b) and 3(c)). Next, Siha and MS751 cell lines were selected for further functional studies.

### 3.4. Knockdown of TAB2 Inhibits CSC-Like Properties in CC

**Figure 4:**
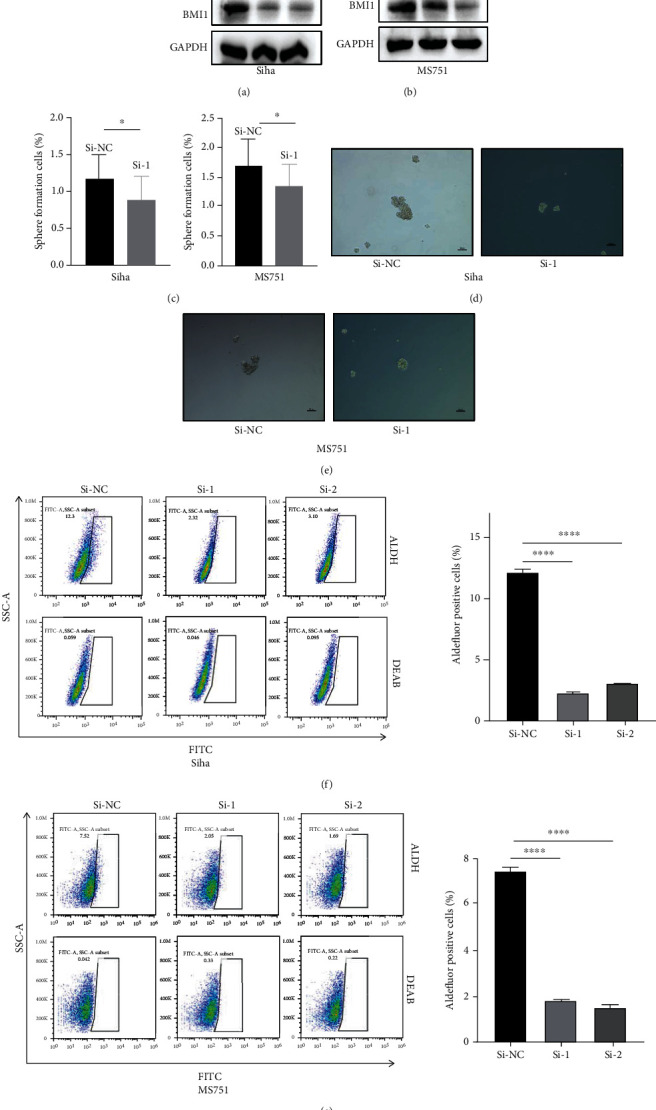
TAB2 knockdown inhibits the stemness of cervical cancer cells. (a) Protein levels of SOX2 and BMI1 were detected by Western blot after knockdown of TAB2 in Siha cells. (b) Protein levels of SOX2 and BMI1 were detected by Western blot after knockdown of TAB2 in MS751 cells. (c) TAB2 knockdown inhibited the sphere formation efficiency in Siha and MS751 cells. (d) TAB2 knockdown inhibited the capacity of sphere formation in Siha cells. (e) TAB2 knockdown inhibited the capacity of sphere formation in MS751 cells. (f) ALDEFLUOR kit was used to assess the proportion of ALDH^br^ in Siha cells intervened with TAB2 or not. (g) The ability of stemness maintenance in MS751 cells was assessed by ALDEFLUOR kit.

Based on the above results, we next investigated the potential influence of TAB2 on CSC-like properties in CC cells. First, we validated the transfection efficacy of TAB2 siRNA at the protein level (Figures 4(a) and 4(b)). Protein levels of stemness-related proteins in CC cells intervened with TAB2 were detected. It is shown that knockdown of TAB2 in Siha and MS751 cells significantly downregulated BMI1 and SOX2. Knockdown of TAB2 in Siha and MS751 cells significantly downregulated sphere-forming efficiency (Figures 4(c)–4(e)). Furthermore, aldehyde dehydrogenase (ALDH) activity, a reliable marker for CSCs, mediated by TAB2 in CC cells was determined [14, 15]. The ALDH activity was significantly reduced in CC cells transfected with si-TAB2 compared with that of negative controls (Figures 4(f) and 4(g)). Thus, we believed that TAB2 was of great significance in maintaining the self-renewal of CC cells.

### 3.5. Knockdown of TAB2 Inhibits *In Vitro* Malignant Phenotypes of CC Cells

**Figure 5:**
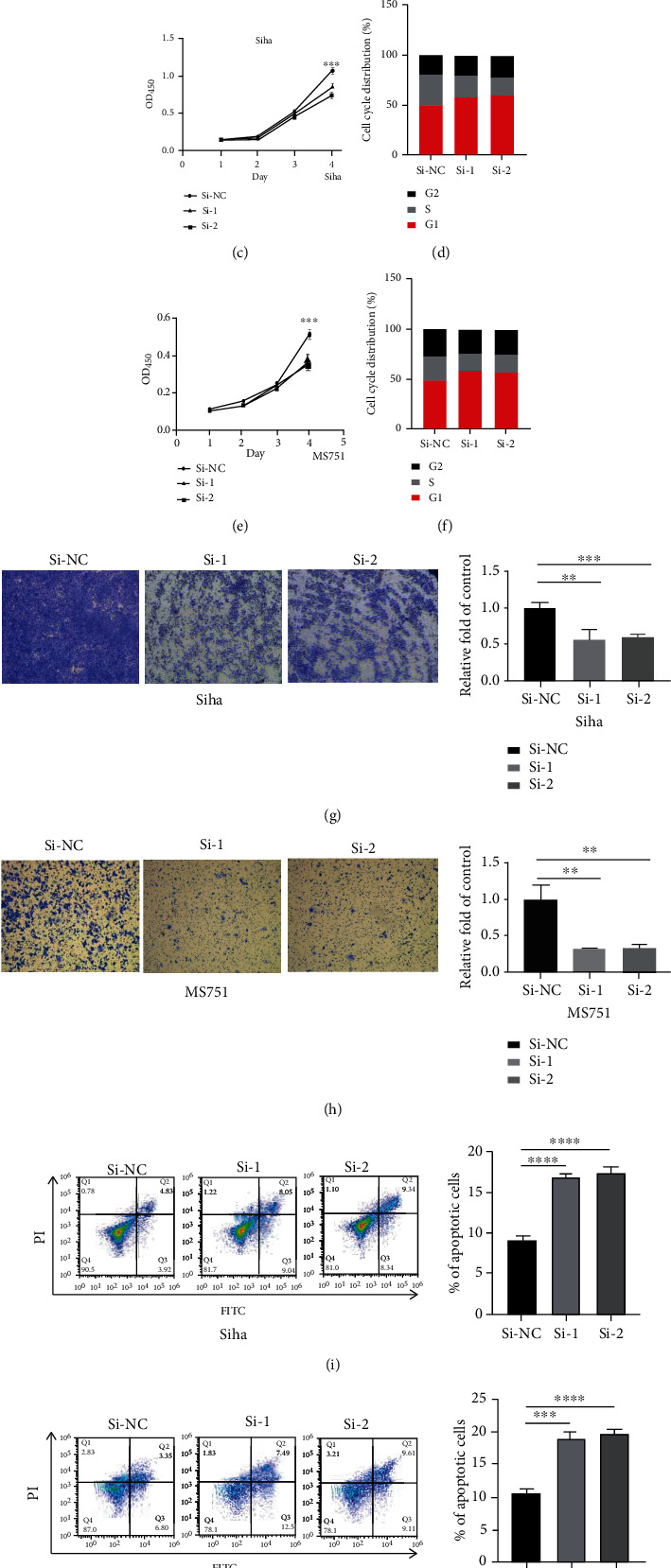
TAB2 knockdown inhibits the biological functions of CC cells. (a) Colony numbers in Siha cells transfected with siTAB2-1, siTAB2-2, or siNC detected by colony formation assay. (b) Colony numbers in MS751 cells transfected with siTAB2-1, siTAB2-2, or siNC detected by colony formation assay. (c) Cell viability of Siha cells transfected with siTAB2-1, siTAB2-2, or siNC detected by CCK-8 assay. (d) Cell cycle distribution of Siha cells transfected with siTAB2-1, siTAB2-2, or siNC detected by the Cell Cycle Kit. (e) Cell viability of MS751 cells transfected with siTAB2-1, siTAB2-2, or siNC detected by CCK-8 assay. (f) Cell cycle distribution of MS751 cells transfected with siTAB2-1, siTAB2-2, or siNC detected by the Cell Cycle Kit. (g) Migration in Siha cells transfected with siTAB2-1, siTAB2-2, or siNC detected by Transwell assay. (h) Migration in MS751 cells transfected with siTAB2-1, siTAB2-2, or siNC detected by Transwell assay. (i) The apoptotic rate of Siha cells transfected with siTAB2-1, siTAB2-2, or siNC detected by flow cytometry. (j) The apoptotic rate of MS751 cells transfected with siTAB2-1, siTAB2-2, or siNC detected by flow cytometry.

Cancer cells are usually manifested as uncontrolled proliferation, avoidance of growth inhibition, resistance to apoptosis, and active invasion and metastasis [16]. To further uncover the functional role of TAB2 in CC cells, we examined proliferation changes in CC cells with TAB2 knockdown through clone formation assay and CCK-8 assay. The results showed that knockdown of TAB2 in Siha and MS751 cells significantly inhibited the ability of clone formation and cell proliferation (Figures 5(a) – 5(c), and 5(e)). The cell cycle distribution of Siha and MS751 cells was detected by flow cytometry, and the results showed that knockdown of TAB2 reduced the ratio of cells distributed at S phase, which was consistent with the inhibited cell growth (Figures 5(d) and 5(f)). Furthermore, Transwell assay demonstrated that knockdown of TAB2 reduced the migratory ability of CC cells (Figures 5(g) and 5(h)). Subsequently, cell apoptosis was identified to be accelerated in CC cells with TAB2 knockdown (Figures 5(i) and 5(j)). In summary, these results indicated that TAB2 exerted a key role in the proliferation, apoptosis, and migration of CC cells.

## 4. Discussion

Existing studies have proven that lncRNAs, miRNAs, and mRNAs regulate the stemness of various types of CSCs [16–18]. Stemness-related pathways are able to alter biological functions of cancer cells like proliferation, migration, invasion, and drug resistance. Presently, the mechanism of CSCs in CC remains largely unknown though many molecules related to stem cell pathways in CC have been identified. In our study, Siha sphere with highly expressed CSC markers was found promoting the rapid progression of CC, showing a prognostic potential in CC patients. Therefore, CC stem cells may be promising therapeutic targets to be applied in clinical practice.

Here, we documented detailed regulating mechanisms of TAB2 in CC stem cells. TAB2 has been shown to play pivotal roles in the activation of the NF-*κ*B pathway through binding to polyubiquitin chains [19]. Importantly, activated NF-*κ*B signaling pathway during tumorigenesis has been well documented [20], which maintains CSC stem-like properties [21]. Consistently, our GSEA of the RNA-seq results showed that upregulated genes in the Siha sphere were significantly related to tumor stemness-related pathways. Because TAB2 is of great importance to regulate cancer stem cells, we analyzed the prognostic potential of it in CC. Data from TCGA database indicated that TAB2 was negatively associated with the overall survival of CC patients. Notably, we found that TAB2 was significantly positively correlated with the mRNA levels of BMI1 and SOX2, which are CSC-related molecules. Previous studies revealed that BMI1 and SOX2 are implicated in the malignant transformation and aggressiveness of multiply tumors [22, 23]. BMI1 participates in the carcinogenesis of CC via upregulating SOX2 [24]. More specifically, we demonstrated that knockdown of TAB2 significantly downregulated BMI1 and SOX2 at mRNA and protein levels, which was consistent with the reduced CSC properties in CC cells with TAB2 knockdown *in vitro*. In the meantime, we found that TAB2 was necessary for mediating a high proliferation rate and a low apoptosis rate in CC cells. Taken together, these results supported the fact that TAB2 promoted the pathogenesis of CC by regulating the CSC stemness pathways. Thereby, our study provided a more profound understanding of TAB2 in CC.

In conclusion, our results reveal the role of TAB2 in regulating the CSC pathway in CC. TAB2 facilitates the progression of CC by activating the stemness-related pathway. Although further research is needed to uncover the carcinogenic mechanisms of TAB2, our current study lays the foundation for developing better treatment strategies in human cancers.

## Data Availability

The data used to support the findings of this study are available from the corresponding author upon request.
